# Deciphering the Material Basis of an Herbal Formula via Integrated Two-dimensional Chromatography, In Silico Screening, and Comparative Pharmacokinetics: A Case Study of Kaiyu Jiangzhuo Formula

**DOI:** 10.1021/acsomega.6c09515

**Published:** 2026-10-29

**Authors:** Nanqi Hou, Qinjing Xia, Zuoyang Li, Xinyue Cao, Zichen Tian, Yuying Zhou, Zepeng Zhang, Kai Yin, Shilei Wang, Dandan Si, Zhuo Man, Xiaolin Tong, Hang Su, Xiangyan Li

**Affiliations:** † Department of Endocrinology, 159345 The First Affiliated Hospital of Changchun University of Chinese Medicine, Changchun 130021, China; ‡ Northeast Asia Research Institute of Traditional Chinese Medicine, Key Laboratory of Active Substances and Biological Mechanisms of Ginseng Efficacy, Ministry of Education, Jilin Provincial Key Laboratory of Bio-Macromolecules of Chinese Medicine, 400878 Changchun University of Chinese Medicine, Changchun 130117, China; § Research Center of Traditional Chinese Medicine, 159346 The First Affiliated Hospital of Changchun University of Chinese Medicine, Changchun, Jilin 130021, China; ∥ 266634 Sciex China, Beijing 100015, China; ⊥ Institute of Metabolic Diseases, Guang’an men Hospital, China Academy of Chinese Medical Sciences, Beijing 100053, China

## Abstract

Kaiyu Jiangzhuo (KYJZ) is a Chinese medicine formula composed of nine herbs. Its efficacy in alleviating glycolipid metabolic disorders and reducing the incidence of diabetes mellitus in overweight patients with prediabetes has been widely validated. Although our previous studies separately characterized the chemical constituents of the KYJZ decoction and the components detected in plasma, the relationship between chemical profiling and systemic exposure remains undefined. In particular, it is unclear which decoction constituents are absorbed into systemic circulation and how obesity modulates their pharmacokinetic profiles. This study aimed to comprehensively profile the components of KYJZ and identify potential bioactive constituents by integrating *in silico* screening, comparative pharmacokinetic, and tissue distribution studies. Two-dimensional liquid chromatography–mass spectrometry (2D-LC–MS) and a hybrid method (data-dependent acquisition/high-resolution multiple reaction monitoring) were employed to decipher the chemical profile and circulating components of KYJZ, respectively. Components exhibiting high binding affinity for antiobesity targets (composite Z-score >0.4) were selected for further pharmacokinetic study. In summary, 316 compounds were identified in KYJZ using 2D-LC–MS/MS. Among these, 99 compounds were recognized as circulating constituents, and 16 with a composite Z-score >0.4 were specifically selected for comparative pharmacokinetic evaluation in mice fed a normal-chow diet (NCD) or a high-fat diet (HFD). Comparative pharmacokinetics revealed that long-term HFD feeding significantly changed the pharmacokinetic behavior of circulating components in mice, especially protoberberine-type alkaloids. Despite their low plasma exposure, they were detected at their highest concentrations in the liver relative to all other tissues examined, suggesting that the liver may represent a relevant organ contributing to the antiobesity effects of KYJZ. Our findings highlight the capability of two-dimensional chromatography to characterize complex herbal medicines and establish a novel analysis pipeline integrating chemical profiling, *in silico* screening, and comparative pharmacokinetic analysis for identifying bioactive components in traditional Chinese medicine formulas.

## Graphical abstract

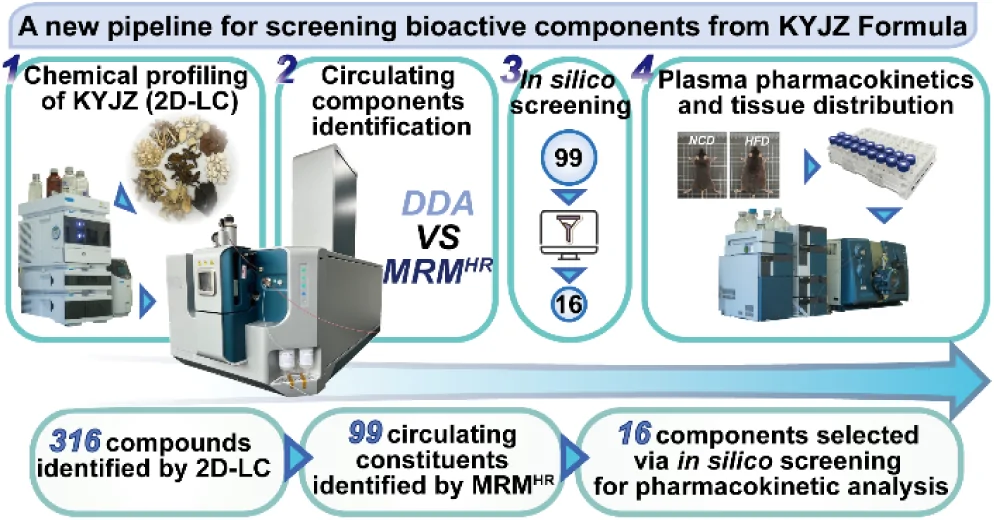

## 1 Introduction

Traditional Chinese medicine (TCM) formulas have a long history of clinical application in the treatment of metabolic diseases such as obesity and diabetes.^1–4^ Despite their extensive clinical use and promising therapeutic outcomes of TCM,^5^ modernization and global acceptance are impeded by the inherent complexity of their material basis. A single herb can contain hundreds of distinct chemical constituents, and a typical TCM formula is a decoction of multiple herbs, resulting in a chemical matrix of immense diversity.^6^ For instance, the Kaiyu Jiangzhuo (KYJZ) formula, composed of nine herbs, has demonstrated efficacy in alleviating glycolipid metabolic disorders and reducing the incidence of diabetes in overweight patients with prediabetes.^7,8^ Although our previous study preliminarily characterized the chemical constituents and circulating components of the KYJZ formula separately, it did not establish the translational link between the chemical profile of the decoction and its *in vivo* exposure.^8^ Therefore, comprehensively identifying the chemical compounds and circulating components, as well as characterizing their pharmacokinetic profiles, is essential for elucidating the pharmacological mechanism of the formula. To achieve these goals, sensitive and robust analytical methods are essential.^9–11^

As a technique for analyzing complex chemical components, multidimensional chromatography can enhance the selectivity and resolution of the separation, thereby increasing the number of identified TCM constituents.^12,13^ This enhancement is fundamentally achieved by using two or more chromatographic columns with distinct separation mechanisms, such as reversed-phase liquid chromatography (RPLC), hydrophilic interaction liquid chromatography (HILIC), or ion exchange chromatography (IEC), which provide complementary selectivities and enable orthogonal separation, thereby increasing peak capacity and the number of identifiable constituents.^14,15^ For instance, in the characterization of *Salvia miltiorrhiza*, a 2D-LC (HILIC-RPLC) system was employed, and 150 compounds were identified, of which 33 compounds were newly discovered.^16^ Similarly, in the characterization of two *Astragalus* species, a three-dimensional LC system (HILIC-RPLC-RPLC) enhanced rapid identification by 71%.^17^ Multidimensional chromatography can effectively separate compounds across a wide polarity range, highlighting its potential use for identifying circulating compounds and evaluating the therapeutic efficacy. In the identification of circulating components, high-resolution multiple-reaction monitoring (MRM^HR^) offers superior sensitivity compared to the traditional data-dependent acquisition (DDA) mode. In this pseudo-targeted strategy, precursor-to-product ion transitions are predefined from the compound library, as in conventional targeted MRM, whereas full high-resolution MS/MS spectra are acquired for each transition, thereby combining the sensitivity and selectivity of targeted acquisition with the structural-confirmation capability of high-resolution mass spectrometry.^18^ This strategy is commonly employed in TCM quality control, mechanistic studies, disease diagnosis, and biomarker discovery.

Pharmacokinetics, which encompasses the absorption, distribution, metabolism, and excretion of substances in the body, plays a crucial role in narrowing hundreds of chemical constituents to a set of potentially active compounds.^19,20^ For a compound to elicit the therapeutic effect following oral administration, it must first undergo absorption from the gastrointestinal tract, survive hepatic first-pass metabolism, enter systemic circulation, and reach target tissues or organs at effective concentrations.^21,22^ Therefore, pharmacokinetic profiling is vital for identifying the active components that are truly circulating in the bloodstream and potentially engaging with treatment targets. Pharmacokinetic profiling for multiherb TCM formulas establishes the relationship between chemical constituents characterized *in vitro* and those reaching systemic exposure *in vivo*. A comprehensive chemical library created by 2D-LC–MS determines absorbable constituents, while pharmacokinetic analysis identifies those entering systemic circulation and reaching target tissues at detectable levels. Connecting formula characterization to potential biological effects, this integrated approach forms the basis of the study’s integrated pipeline.

Both physiological states caused by diseases and the chemical scaffolds of compounds influence pharmacokinetic processes and *in vivo* biodistribution.^23,24^ A study on the Zhimu–Huangbai herb pair revealed that the levels of seven major bioactive components differed between normal rats and those with type 2 diabetes.^25^ Chemical scaffolds represent another critical factor governing pharmacokinetics, especially concerning absorption through the small intestine.^24^ Previous research found that flavonoids can be absorbed by the small intestine of rats in the form of glycosides.^26^ Overall, these studies demonstrated that the body’s handling of TCM constituents is dynamically influenced by both the disease states and the chemical scaffolds of compounds.

The present study was designed to expand upon our previous studies by conducting a more comprehensive and in-depth analysis of the potential bioactive components of the KYJZ formula. By integrating 2D-LC-based chemical profiling and pharmacokinetic analysis, we aimed to comprehensively characterize the chemical constituents present in the KYJZ formula and identify the bioactive components that are absorbed into the systemic circulation following oral administration. This novel TCM study pipeline is set up to characterize pharmacokinetic parameters and provide reliable quality markers to support the clinical application of the KYJZ formula in treating obesity. Furthermore, this research might facilitate the discovery of novel therapeutic agents derived from this traditional remedy.

## 2 Materials and Methods

### 2.1 Chemicals and Reagents

The nine traditional Chinese medicines constituting the KYJZ formula (Tongrentang Pharmacy Co., Ltd., Beijing, China) were authenticated by the Department of Pharmacy, the Affiliated Hospital of Changchun University of Chinese Medicine (Changchun, China), according to the Chinese Pharmacopoeia (2020 edition). Detailed information on the KYJZ formula is listed in Table S1. Acetonitrile (A998-4), methanol (A452-4), formic acid (A117-50), and acetic acid (A113-50), all of which were HPLC grade, were purchased from Thermo Fisher Scientific Co., Ltd. (Waltham, MA, USA). The details of standard compounds and two internal standards (ISs) used in this study are presented in Table S2. Ultrapure water was supplied by Wahaha Co., Ltd. (Hangzhou, China).

### 2.2 Chemical Profiling of KYJZ

#### 2.2.1 KYJZ Sample Preparation for Chemical Profiling

The nine herbs of KYJZ were weighed according to the specified mass ratio (Table S1) and decocted twice with 10 volumes (mL/g) of water for 40 min each time. Then, the combined supernatant was filtered, concentrated, and lyophilized to obtain the KYJZ powder. To prepare the KYJZ analysis solution, 50 mg of KYJZ powder was accurately weighed and dissolved in 50 mL of an 80% (v/v) methanol in water. Then, the resulting mixture was sonicated for 40 min and centrifuged to collect the supernatant for the first-dimensional (^1^D) chromatographic separation. After 50 runs of the ^1^D chromatographic separation, each fraction was dried under a stream of N_2_ and reconstituted in 1 mL of 80% (v/v) methanol for the second-dimensional (^2^D) chromatographic separation.

#### 2.2.2 Stationary Phase Screening and Orthogonality Assessment of 2D Separation

The ^1^D separation compared two IEC columns, namely, Venusil SCX (Agela, Tianjin, China) and PhenoSphere SAX (Phenomenex, Torrance, CA, USA). Subsequently, the ^2^D separation screened four reversed-phase columns, including Aq-C18 (Agilent, Santa Clara, CA, USA), EC-C18 (Agilent), Epic-C18 (PerkinElmer, Waltham, MA, USA), and HSS-T3 (Waters, Milford, MA, USA), under optimized LC–tandem mass spectrometry (MS/MS) conditions. The details of all columns are provided in Table S3. The ^1^D separation of KYJZ was performed on an Agilent 1260 Infinity II HPLC system (Agilent). The mobile phase consisted of 0.1% formic acid in water (A) and acetonitrile (B), and the following isocratic program was applied at 90% (B) for 15 min. The flow rate was 1 mL/min, and the column temperature was 25 °C. The wavelength of the diode array detector was 210 nm. The ^2^D separation was performed on an Exion LC AE ultraperformance liquid chromatography (UPLC) system (SCIEX, Framingham, MA, USA) maintained at a temperature of 40 °C. The mobile phases consisted of 0.05% formic acid in water (A) and acetonitrile (B), and the following gradient program was applied: 0–1 min, 5% B; 1–2 min, 5%–15% B; 2–15 min, 15%–26% B; 15–16 min, 26%–33% B; 16–21 min, 33%–45% B; 21–26 min, 45%–65% B; 26–29 min, 65%–98% B; 29–33 min, 98%–98% B; 33–33.1 min, 98%–5% B; and 33.1–37 min, 5% B. The UPLC system and chromatographic conditions used in the pharmacokinetic study were identical to those of the ^2^D separation of KYJZ.

The orthogonality of the 2D system was quantified as the A_o_ value, calculated using the asterisk equations proposed by *Camenzuli* and *Schoenmakers*.^27^ This metric quantifies the distribution of peaks across the 2D separation space and serves as a correction factor for theoretical peak capacity. A higher A_o_ indicates greater orthogonality, meaning that the two dimensions provide complementary selectivity and a larger portion of the theoretical peak capacity is practically usable. Retention times of 26 reference standards were acquired on each candidate column to calculate the A_o_ values for all possible column pair combinations.

#### 2.2.3 Mass Spectrum Acquiring Method for the Chemical Profiling of KYJZ

A quadrupole time-of-flight (Q-TOF) 7600 mass spectrometer (SCIEX) equipped with an electrospray ionization (ESI) source was used for the chemical profiling of KYJZ. The MS parameters were optimized based on the instrument manufacturer’s recommendations and previously published methods.^28^ The optimized MS parameters were as follows: ion source temperature, 500 °C; ion spray voltage, 5500 V (positive)/4500 V (negative); gas 1, 50 psi; gas 2, 50 psi; curtain gas, 35 psi; CAD gas, 9; declustering potential, 80 V; collision energy, 35 ± 15 V; an *m*/*z* range of 80–1500 with an accumulation time of 0.15 s for TOF MS, and an *m*/*z* range of 50–1500 with an accumulation time of 0.015 s for TOF MS/MS. Data acquisition combined DDA and dynamic background subtraction modes. To build a chemical library, the chemical information on the nine herbs in KYJZ was obtained from the SCIEX OS database and the existing literature.^29–36^ The precursor ions, product ions, and ion adduct modes were recorded to establish a compound library of KYJZ. Then, the acquired data were matched against the KYJZ literature-based compound library with the scoring criteria of mass error ≤ 5 ppm and isotope deviation ≤ 5%.

### 2.3 Experimental Animals

For the pharmacokinetic study, 25 male C57BL/6J mice (aged 6 weeks, weighing 18–22 g) were purchased from Yisi Experimental Animal Technology Co., Ltd. (Changchun, China) and housed at Changchun University of Chinese Medicine. The animals were maintained under controlled conditions (temperature, 20–24 °C; humidity, 40%–60%; 12 h/12 h light/dark cycle) with ad libitum access to standard food and water. After a week of acclimation, they were divided into the NCD (n = 7) and HFD (n = 18) groups. All animal experiments were conducted with the approval of the Experimental Animal Ethics Committee of Changchun University of Chinese Medicine (Approval No. 2024005) and in accordance with the Guide for the Care and Use of Laboratory Animals. Mice in the NCD group received a standard diet, whereas those in the HFD group were fed an HFD (60% fat, Research Diets, New Brunswick, NJ, USA, D12492). Feeding was conducted in both groups for 12 weeks. Obesity was considered successfully induced when the body weight of the HFD group exceeded that of the NCD group by 20%. The obese mice were then subdivided into two groups for the pharmacokinetic (n = 6) and tissue distribution studies (n = 12). In the pharmacokinetic and tissue distribution studies, an oral dose of 2.1 g/kg of KYJZ was required, whereas 4.2 g/kg was required for the identification of circulating components. The 2.1 g/kg dose administered to mice was converted from the clinically effective dosage for humans, and the dose was doubled to identify more circulating components.^8^

### 2.4 Circulating Constituent Identification

A 100-μL aliquot of plasma was collected from control mice at 0.5, 2, and 6 h after oral administration (KYJZ, 4.2 g/kg). The samples were collected via retro-orbital venous plexus puncture.^37^ Then, 400 μL of methanol:acetonitrile (1:1), which contains the ISs (50 ng/mL tetrahydropalmatine and 5 ng/mL 6-chloroflavone), was added into samples to precipitate proteins. The mixtures were vortexed for 1 min, sonicated in an ice-water bath for 15 min, and then refrigerated at −20 °C for 1 h. The supernatant was evaporated to dryness under vacuum condition at 0 °C using a low-temperature freeze concentrator (JM CV200, Beijing, China) at 1,300 rpm. The resulting residue was reconstituted in 80% methanol to identify the circulating constituents. The prepared samples were identified in the DDA and MRM^HR^ modes on Q-TOF 7600 to identify the stable circulating components of KYJZ.

DDA: The mass spectrum acquiring parameters of DDA were the same as those for the ^2^D separation.

MRM^HR^: MRM^HR^ had the same TOF-MS parameters as DDA, and the precursor and product ions for the 469 transitions are listed in Table S4, with an accumulation time of 0.015 s for each transition.

### 2.5 In Silico Screening Method for Antiobesity Drug Candidate Discovery from Circulating Compounds

To build a comprehensive quantitative evaluation system of antiobesity, we established integrating two composite score system including two aspects, lipid homeostasis regulation ability, and blood glucose regulation. For evaluating lipid homeostasis regulation ability, we summarized FDA-approved small-molecule drug targets, including FFAR1 (PDB ID: 5TZR), FFAR4 (PDB ID: 8H4K), PPARα (PDB ID: 2P54), PPARγ (PDB ID: 3NOA), and PPARδ (PDB ID: 3OZ0). And then, we run the parallel molecular docking by deep learning framework-based molecular docking program GNINA v1.1.^38^ The details are described as follows. The crystal structure of the target was retrieved from the RCSB Protein Data Bank. The receptor was prepared by removing water molecules, ions, and cocrystallized ligands, retaining only the protein chain. Hydrogens and partial charges were assigned using Open Babel 3.1.0,^39^ and the structure was converted to the PDBQT format. The binding site was defined based on the coordinates of the cocrystallized ligand. And then, a total of 99 circulating compounds were prepared from their SMILES representations using RDKit. Three-dimensional conformers were generated using the ETKDGv3 algorithm, followed by MMFF94 force field optimization. Molecular docking was performed using GNINA v1.1, employing a convolutional neural network (CNN) scoring model, with an exhaustiveness of 8 and a maximum of 9 binding poses per ligand. Compounds were ranked by the best CNN affinity score across all returned poses. All docking calculations were performed on CPUs in parallel. Similarly, α-glucosidase (PDB ID: 3TOP), DPP-4 (PDB ID: 5T4F), ABCC8 (PDB ID: 6JB3), GLUT4 (PDB ID: 7WSM), and SGLT2 (PDB ID: 7VSI) were selected to evaluate glucose regulation.

The docking protocol was validated by redocking the cocrystallized ligand of each target into its prepared binding site using the identical GNINA v1.1 settings (exhaustiveness = 8; 9 poses). The cocrystallized ligands were MK-8666 (5TZR), GW9508 (8H4K), GW735 (2P54), 5BC (3NOA), 3OZ (3OZ0), acarbose (3TOP), ligand 34p (5T4F), repaglinide (6JB3), cytochalasin B (7WSM), and empagliflozin (7VSI). Heavy-atom RMSD values between redocked and crystallographic poses were computed with spyrmsd using symmetry-corrected matching.^40^

### 2.6 Quantitative Evaluation of Molecular Docking Activity

To comprehensively assess the binding potential of 99 compounds across multiple therapeutic targets, a quantitative composite scoring system was developed, integrating molecular docking results from two target data sets: lipid-regulation targets (FFAR1, FFAR4, PPARα, PPARγ, PPARδ) and antidiabetic targets (ABCC8/SUR1, DPP-4, GLUT4, SGLT2, α-glucosidase). For each compound and its corresponding target, three docking metrics were recorded, including Vina score (kcal/mol), CNN score, and CNN affinity. The Vina score in AutoDock Vina is an empirical assessment of binding free energy. A more negative value denotes stronger binding.^41^ In contrast, GNINA uses the CNN score, which ranges from 0 to 1, as a pose-classification probability to order poses. Additionally, CNN affinity indicates the CNN-predicted binding affinity in p*K* units (−log K), with higher values representing more potent binding.^41^ Prior to normalization, the Vina score was sign-inverted to ensure that higher values consistently represent stronger binding in all metrics. The Z-score for each of the 30 resulting target-metric combinations was calculated using the formula Z = (x – μ)/σ. Here, μ and σ represent the mean and standard deviation of the metric across all 99 compounds for the specific target. This standardization process was conducted within each target-metric combination and not across the 10 targets. Data set-specific composite scores (Lipid Composite Z and Diabetes Composite Z) were calculated as the mean Z-score across the 15 metrics within each data set. An Overall Composite Z-score, representing the mean of all 30 standardized metrics, was used to rank compounds comprehensively, with higher scores indicating superior antiobesity drug target.

The three metrics (Vina score, CNN affinity, and CNN score) were equally weighted a priori because no experimental binding data were accessible to derive supervised weights. These metrics are complementary in design, comprising an empirical free energy estimate (Vina score), a deep-learning affinity prediction (CNN affinity), and a pose-classification probability (CNN score).

### 2.7 Concentration–Time Curve Analysis and Tissue Distribution of KYJZ in Mice

On the basis of the identification of circulating constituents in mice, 16 compounds were selected as target analytes for pharmacokinetic analysis. Each analyte was prepared as a 1 mg/mL stock solution in 80% methanol, and then, the stock solution of each analyte was mixed into a stock solution of appropriate concentration for the subsequent analysis.

A 100-μL aliquot of plasma was collected from the NCD (n = 6) and HFD groups (n = 6) at 0.17, 0.5, 1, 2, 4, 6, 8, 12, 24, 36, 48, and 72 h after oral administration (KYJZ, 2.1 g/kg). The sample processing method was the same as that in Section 2.4, but the supernatant was not mixed after the first centrifugation.

After oral administration of 2.1 g/kg KYJZ, 12 HFD-fed mice were randomly euthanized at 0.5, 2, 6, and 12 h (n = 3/group). Tissues (liver, spleen, lungs, kidneys, and heart) were immediately collected and stored at −80 °C for tissue distribution analysis. The sample preparation process is presented in Supplementary Method 1.

A 6500+ triple quadrupole mass spectrometer (SCIEX) equipped with an ESI source was used in the pharmacokinetic and tissue distribution analyses. For each compound, MRM transitions were optimized in both positive- and negative-ionization modes, and the mode with higher signal intensity was selected for quantification to ensure maximal sensitivity. The MS parameters were the same as those in Section 2.2.3.

### 2.8 Method Validation

Validation of the established methods, including selectivity, accuracy, precision, linearity, stability, matrix effects, and recovery, was conducted following the bioanalytical method validation guidance of the US Food and Drug Administration (FDA, CDER, 2018). The process is presented in Supporting Information Method 2.

### 2.9 Statistical Analysis

All quantitative analyses were processed using SCIEX OS 3.4, and pharmacokinetic parameters including the maximum plasma concentration (*C*_max_), terminal elimination half-life (*t*_1/2_), time to reach *C*_max_ (*T*_max_), and area under the plasma concentration–time curve (AUC_0‑t_) were analyzed via the noncompartmental model in WinNonlin 8.1.

The results obtained from the pharmacokinetic study are presented as the mean ± standard deviation (SD). Statistical significance between two groups was analyzed by unpaired Student’s *t* test. For multiple group comparisons, one-way analysis of variance was employed. All analyses were performed using GraphPad Prism (GraphPad Software, Boston, MA, USA). Analyzed results with *P* < 0.05 were considered statistically significant.

## 3 Results and Discussion

### 3.1 Chemical Profiling of KYJZ

#### 3.1.1 Establishment of a 2D-LC System

Initially, we analyzed the chemical composition of KYJZ using 2D-LC–MS techniques and established a chemical library for all identified compounds. The column, mobile phase, additives, and gradient program of ^2^D chromatography were optimized by one-factor experiments to improve separation performance. Huanglian, the main herb of KYJZ, is known as the “emperor” herb in TCM theory, and it was previously reported to be rich in alkaloids such as berberine, palmatine, and coptisine, which have antiobesity effects.^42,43^ Ganjiang, one of the main herbs of KYJZ, contains numerous acidic phenolic compounds such as ferulic acid, 6-gingerol, and 8-gingerol.^30,44^ Therefore, we evaluated two IEC columns known for their superior performance in separating ionic compounds and alkaloids, which were strong cation exchange (SCX) and strong anion exchange (SAX) columns, as potential choices for ^1^D chromatographic separation.^45,46^ First, we compared the separation performance of SCX and SAX columns under the same elution conditions. Under the same conditions, the SCX column separated 11 peaks, whereas the SAX column separated only four peaks, indicating that the SCX column has greater peak capacity than the SAX column (Figure S1A–B). Therefore, the SCX column was selected for the ^1^D separation of compounds in KYJZ.

The elution gradients, mobile phases, and additives were optimized using the SCX column. For the organic phase, different ratios of methanol and acetonitrile were compared. The best peak capacity for KYJZ was achieved when the acetonitrile concentration was 90% (Figure S1A–B). After comparing different organic phases, the effect of different aqueous phase additives was assessed. The peak shapes obtained using 0.05% formic acid and 0.05% acetic acid in the aqueous phase were not significantly different (Figure S1C). However, the peak at 4.5 min separated into two peaks when the concentration of formic acid was increased to 0.1%, indicating greater peak capacity. Thus, 0.1% formic acid in water was selected as the final aqueous phase (Figure S1D). The results of ^1^D separation of KYJZ are presented in Figure 1A. Full wavelength scanning (190–400 nm) revealed that the KYJZ constituents exhibited maximal UV absorption at 210 nm (Figure 1B).

**Figure 1:**
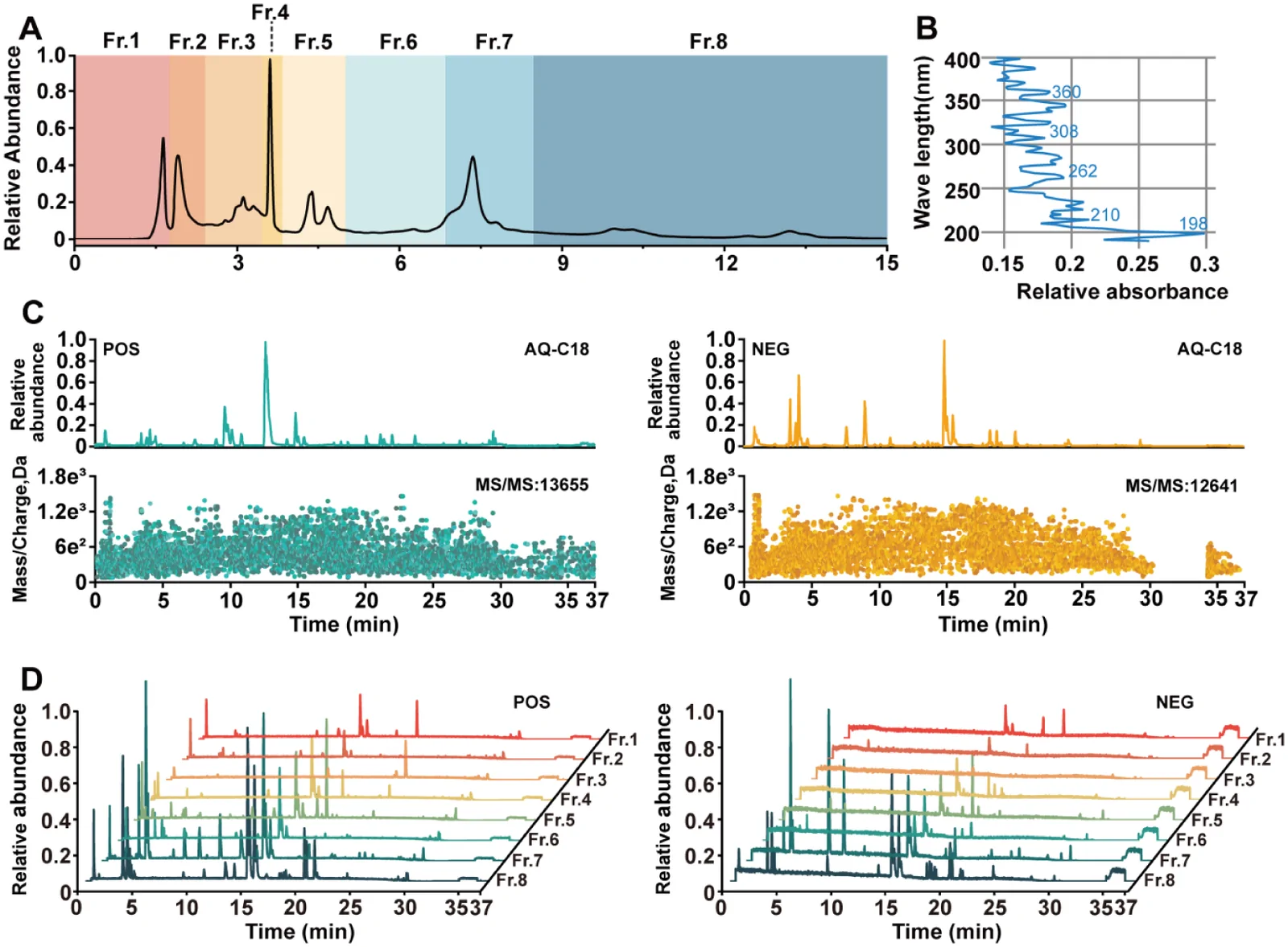
Optimization of 2D chromatography. UV absorption spectra of the first dimension separation at 210 nm (A). Full-wavelength (190–400 nm) UV chromatograms of the first-dimension separation (B). The TIC and MS^2^ spectrum number of KYJZ under Aq-C18 (C). The second-dimensional TIC of the eight fractions obtained by first-dimensional separation (D).

Then, four reversed-phase columns were selected to screen the ^2^D stationary phases by comparing the peak capacity and shapes alongside the number of MS/MS spectra under the same elution gradient. The Aq-C18 column yielded a higher number of MS/MS spectra and a better peak shape than the other columns, and thus, this column was selected for the ^2^D stationary phase (Figure 1C). The results obtained with the other three columns are provided in Figure S1. Subsequently, each of the eight fractions was analyzed by Q-TOF-MS in both positive- and negative-ionization modes. The resulting total ion chromatogram (TIC) is shown in Figure 1D.

#### 3.1.2 Evaluation of Orthogonality

The orthogonality of the established 2D chromatographic separation system was evaluated using the asterisk equations.^27^ The retention times and normalized retention times of the 26 reference standards in ^1^D and ^2^D separations are summarized in Table S5. On the basis of the asterisk equations, four Z parameters (Z_–_, Z_+_, Z_1_, and Z_2_) and A_o_ were calculated for all column combinations investigated (Table S6). Among these, the combination of an SCX column in the ^1^D and an Aq-C18 column in the ^2^D yielded the highest A_o_ value of 0.79, indicating excellent separation orthogonality between the two dimensions (Figure S2). These results confirm the effectiveness of the established 2D-LC method in characterizing the chemical constituents of KYJZ.

#### 3.1.3 Systematic Characterization of KYJZ

The eight fractions separated by ^1^D chromatography were analyzed by Q-TOF-MS (Figure 1D). In total, 241 and 316 chemical constituents were identified by 1D and 2D chromatographic analyses, respectively, following screening and deduplication (Table S7). Among these, twenty-eight compounds were confirmed with authentic reference standards, whereas others were identified by MS/MS fragmentation patterns. Using 2D chromatography, we identified a significantly larger number of compounds than the 52 compounds reported in our previous study.^8^ Thus, 2D chromatography techniques significantly improved the characterization of the chemical composition of KYJZ. Using mangiferin as an example, marked differences were observed in its MS/MS spectra between one-dimensional (1D) and two-dimensional (2D) LC–MS analyses. In the 1D analysis of KYJZ, no MS/MS spectra were acquired for mangiferin at its retention time (Figure 2A). However, following 2D chromatographic separation, the number of acquired MS/MS spectra increased substantially at its corresponding retention time (Figure 2B), and these MS/MS spectra were consistent with those obtained from the reference standards (Figure 2C,D). The results indicate that two-dimensional chromatography significantly improves the quality of the MS/MS spectra. Collectively, these findings demonstrate that 2D chromatography can be used to build comprehensive chemical libraries of TCM formulas by overcoming the insufficient separation ability of 1D chromatography.^9^

**Figure 2:**
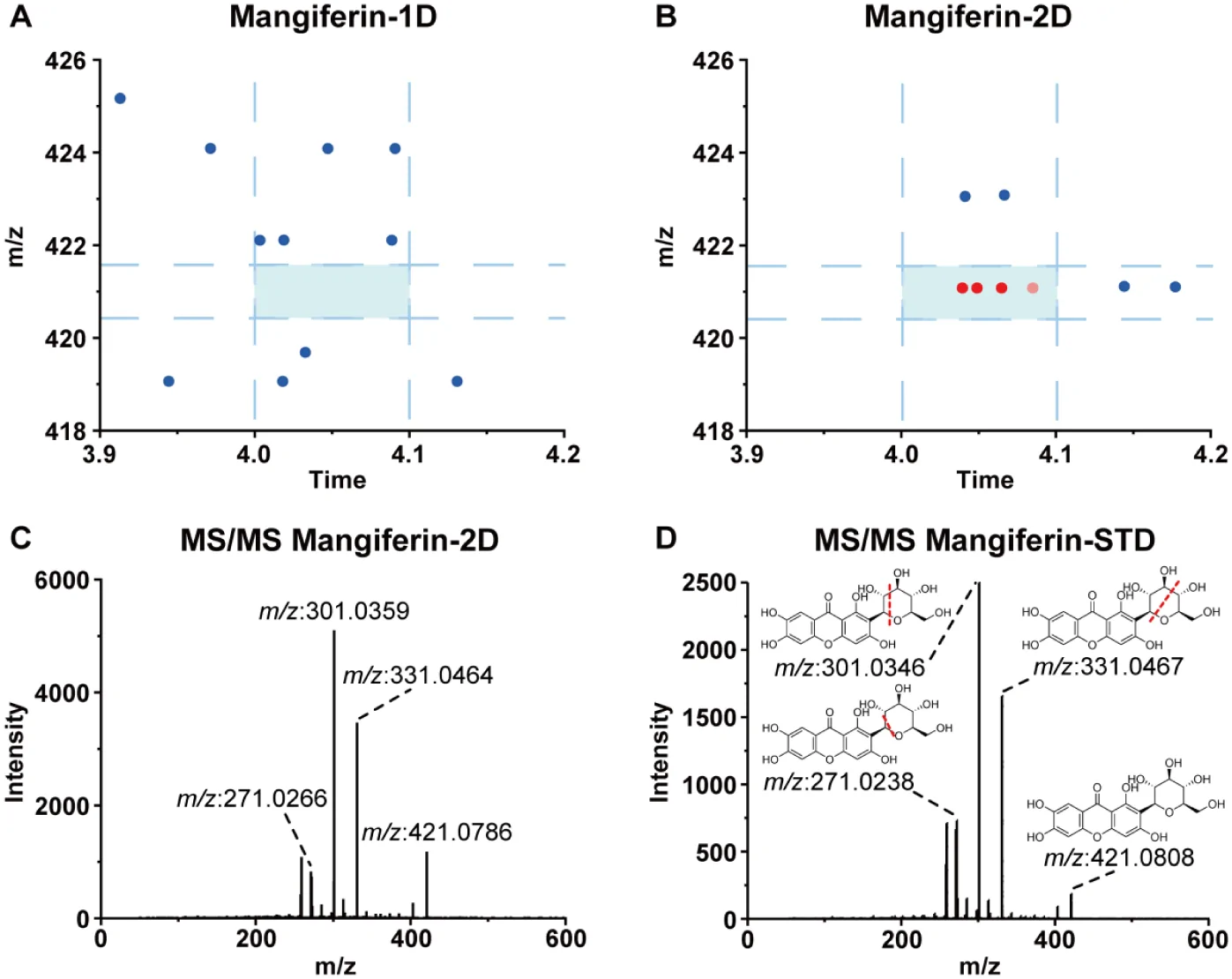
Different MS/MS spectra between 1D and 2D chromatography. Acquisition points of mangiferin in 1D chromatography (A). Acquisition points of mangiferin in 2D chromatography (B). MS/MS spectra of mangiferin in 2D chromatography (C). MS/MS spectra of mangiferin in the standard reference (D).

### 3.2 Circulating Constituent Identification

Owing to the complexity of chemical components in TCM, pharmacokinetic analysis is a crucial method for identifying potential bioactive compounds.^47^ After chemical profiling of KYJZ, we conducted a pharmacokinetic study, including identification of the circulating constituents, pharmacokinetic profiling of these constituents, and an assessment of their tissue distribution. This helped to reveal the levels of potential bioactive constituents of KYJZ in the target tissues. For the comprehensive identification of circulating constituents, a high dose of 4.2 g/kg was orally administered to mice. This was aimed to maximize the number of detectable circulating constituents and establish a comprehensive *in vivo* constituent profile. For the subsequent quantitative pharmacokinetic study, a dose of 2.1 g/kg, which was converted from the clinically effective dosage for humans, was selected to ensure that the derived pharmacokinetic parameters are pharmacologically relevant. We acknowledge that systemic exposure may not increase linearly with dose; therefore, we do not assume that all 99 constituents detected at 4.2 g/kg achieve a meaningful exposure at 2.1 g/kg. While the pharmacological relevance of the identified constituents remains to be established in subsequent efficacy studies, the present work provides a comprehensive foundation for prioritizing candidate compounds from the vast chemical space of KYJZ for future mechanistic investigations.

To characterize the circulating constituents in plasma, two different MS methods (DDA and MRM^HR^) were compared after the oral administration of KYJZ (4.2 g/kg). The peak abundances for high- (10^5^ < cps <10^6^), medium- (10^4^ < cps <10^5^), and low-response (10^3^ < cps <10^4^) transitions in the positive and negative ionization modes by MRM^HR^ are presented in Figure 3A and B, respectively. Meanwhile, the relative abundances for DDA in these modes are presented in Figure 3C and D, respectively. In mass spectrometry, signal intensity reflects both analyte abundance and ionization efficiency, which varies across chemical scaffolds. Thus, signal intensity is not a surrogate for molar concentration. However, in data-dependent acquisition, high-response precursors are preferentially fragmented, providing richer structural information for identification. For subsequent pharmacokinetic analysis, adequate signal intensity is equally critical. Analytes near the lower limit of quantification compromise MRM transition development, peak integration, and pharmacokinetic parameter estimation.^48^ The results showed that the number of identified stable circulating constituents (cps >1000) was 65 for DDA and 99 for MRM^HR^. Importantly, MRM^HR^ detected all circulating constituents identified by DDA (Figure S3A). The results indicated that when detecting low-response analytes, MRM^HR^ is more sensitive compared to DDA.^49^ In the DDA mode, setting the number of candidate product ions (MS/MS) to 30 could lead to undetected product ions of low-abundance compounds because of coelution.^9,50^ The use of different MRM transitions in the MRM^HR^ mode enables the discrimination of compounds with identical retention times. Furthermore, scheduled MRM^HR^ monitors each MRM transition only within a defined retention time window, which helps prevent the misassignment of isomers sharing identical product ions. For example, berberine and epiberberine have the same molecular formulas and characteristic product ions. Even though they exhibit different retention times in the standard reference, the data-processing software could still mismatch these compounds due to their identical characteristic product ions. This issue can be resolved by employing MRM^HR^ based on the scheduled scan time.

**Figure 3:**
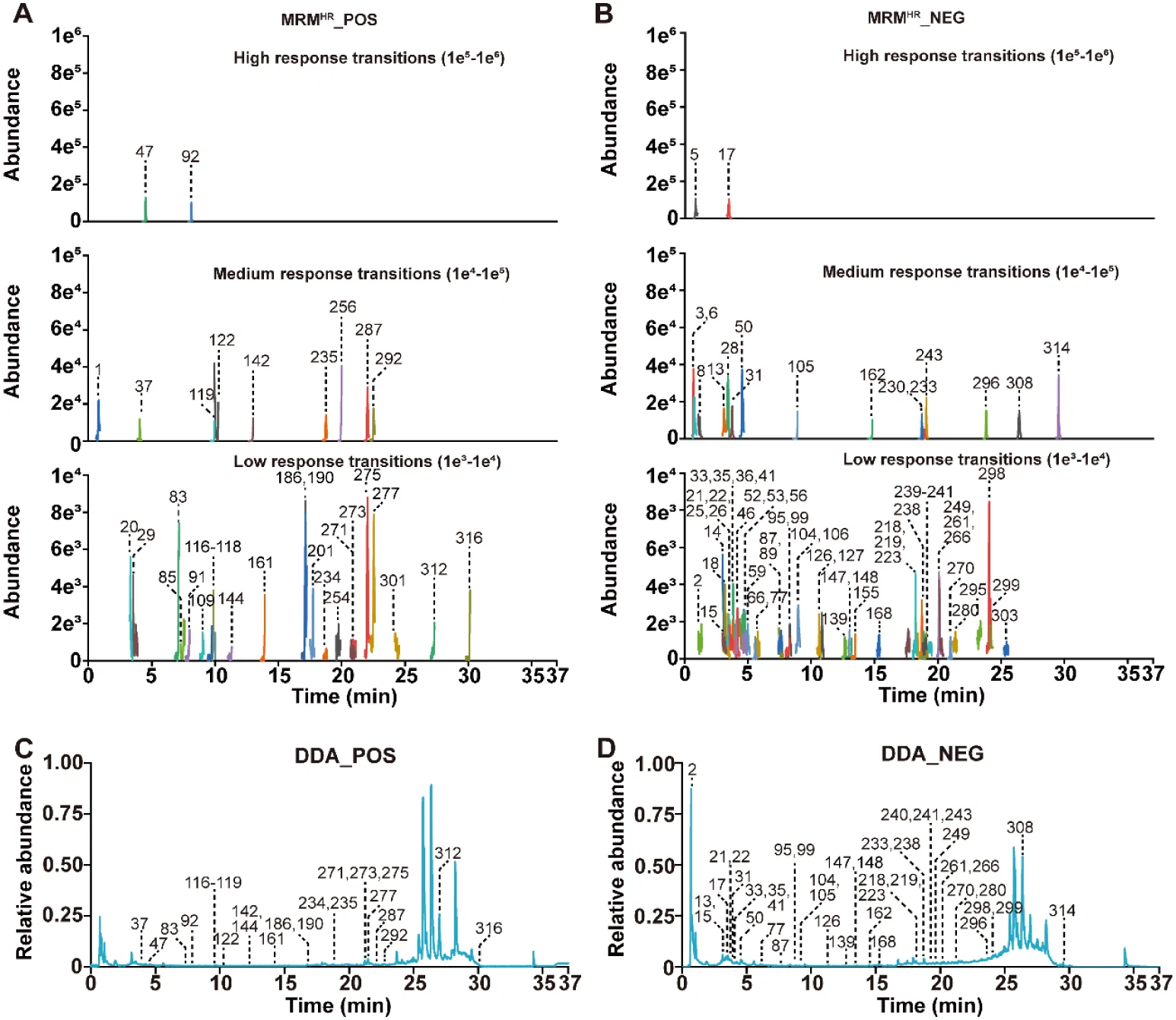
Identification of circulating components by two mass spectrometry methods. Circulating components identified by MRM in positive (A) and negative (B) ionization modes. Compounds were categorized by response intensity: high (1e^5^ – 1e^6^ cps), medium (1e^4^ – 1e^5^ cps), and low (1e^3^ – 1e^4^ cps) response transitions. Circulating components identified by DDA in positive (C) and negative (D) ionization modes.

The circulating constituents detected by MRM^HR^ included 13 alkaloids, 8 anthraquinones, 3 fatty acids, 24 flavonoids, 11 glycosides, 6 phenolic acids, 2 phenylpropanoids, 23 triterpenes, 1 lignan, and 8 others (Figure S3B, Table S7).

### 3.3 Identification of Antiobesity Candidates from Circulating Components via Multitarget In Silico Screening

To screen potential antiobesity compounds, we performed the high-throughput virtual screening of 99 circulating components against ten targets involved in lipid homeostasis and glucose regulation (Table S8). Cocrystallized ligands were redocked to validate the reliability of the docking results (Figure S4, Table S9). These components were then assessed using three docking metrics, including Vina score (kcal/mol), CNN score, and CNN affinity, to generate a composite Z-score (Table S10). The composite Z-score is a relative, within-data set ranking metric intended for prioritization rather than an absolute predictor of pharmacological activity.

On the basis of this comprehensive ranking rule, 16 circulating components with a composite Z-score of >0.4, namely, berberine, coptisine, epiberberine, jatrorrhizine, magnoflorine, palmatine, naringin, hesperidin, timosaponin A3, mangiferin, neomangiferin, ginsenoside Rb1, 6-gingerol, 8-gingerol, chrysophanol, and aloe-emodin, were selected for subsequent pharmacokinetic analysis (Figure 4). In our data set, a Z-score threshold of >0.4 selected approximately 15% of the circulating components of KYJZ. This threshold was chosen to balance statistical stringency with practical feasibility for the downstream pharmacokinetic analysis. Sensitivity analysis across alternative thresholds (Figure S5, Table S11) showed fully nested candidate sets: all 16 candidates were retained at Z > 0.2 (n = 39) and Z > 0.3 (n = 29), while all compounds selected at Z > 0.5 (n = 9) and Z > 0.6 (n = 3) were contained within the original 16. A top-20 cutoff also recovered all 16 candidates.

**Figure 4:**
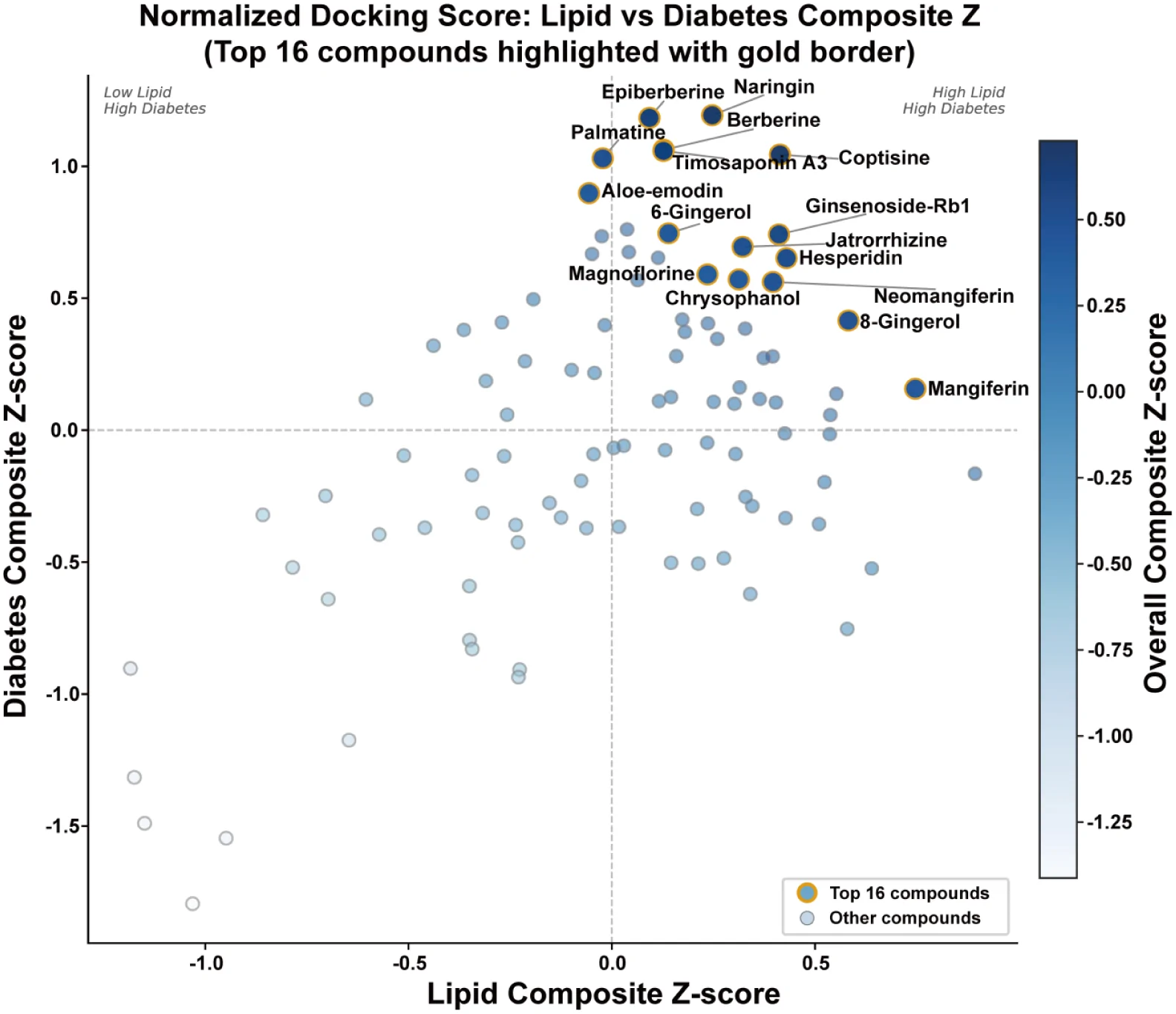
Identification of 16 potential bioactive components by *in silico* screening.

Among the 990 ligand-target pairs analyzed, the pairwise correlations between the three metrics were found to be weak (Spearman ρ = 0.31, 0.20, and 0.08 for Vina score-CNN affinity, Vina score-CNN score, and CNN score-CNN affinity, respectively; see Figure S6A, Table S12). This indicates that the contributions are largely nonredundant. Adjusting the weights moderately (2:1:1 or 1:1:2) had minimal impact on the ranking (ρ = 0.96 compared to the equal-weighted ranking, with 12/16 and 14/16 of the original candidates remaining in the top 16). In contrast, rankings based on a single metric showed significant deviations (ρ = 0.80, 0.62, and 0.21 for CNN affinity, Vina score, and CNN score alone; refer to Table S13, Figure S6B). Consequently, the composite score combines diverse information rather than relying on any individual metric. By integrating multitarget affinity metrics, this screening approach reveals the therapeutic potential of circulating components, offering a more comprehensive screening strategy than conventional single-target approaches.

However, molecular docking scores are hypothesis-generating and do not constitute evidence of actual biological activity. In the absence of experimental binding assays, the 16 candidates prioritized here should be regarded as potential bioactive constituents that require further experimental confirmation.

### 3.4 Method Validation

For the remaining 16 analytes, the characteristic product ion fragments were obtained using a 6500+ mass spectrometer for the MRM method development (Table 1). Method validation was performed for the 16 analytes using UPLC-MS/MS. By comparison of blank plasma samples and plasma samples obtained after administering KYJZ at different concentrations, no significant endogenous or exogenous interference was detected (Figure S7). Calibration curves were plotted as peak area (x) versus concentration (y), and all curves displayed good linearity, with correlation coefficients (R^2^) greater than 0.99 (Table 2). The lower limit of quantification for each curve, as well as the analyzed concentration range, is presented in Table 2.

**Table 1:** Optimized MRM Detection Parameters for 16 Analytes and 2 Internal Standards by UHPLC-MS/MS

Analytes	Ion mode	Precursor ion (*m*/*z*)	Product ion (*m*/*z*)	Declustering potential (V)	Collision energy (V)
Hesperidin	[M – H]^−^	609.26	301.02	–73	–33
Naringin	[M – H]^−^	579.16	271	–175	–45
Ginsenoside Rb1	[M – H]^−^	1107.8	945.7	–157	–65
Mangiferin	[M – H]^−^	421.08	300.98	–80	–30
Timosaponin A3	[M + HCOO]^−^	785.47	739.48	–90	–30
Aloe-emodin	[M – H]^−^	269.01	240.03	–110	–30
Chrysophanol	[M – H]^−^	253.08	225.01	–120	–36
Neomangiferin	[M – H]^−^	583.17	331.01	–160	–48
6-Gingerol	[M + H]^+^	295.21	177.08	110	14
8-Gingerol	[M + H]^+^	323.21	137.05	130	32
Berberine	[M]^+^	336.2	292.2	50	70
Coptisine	[M]^+^	320.2	292.1	80	40
Epiberberine	[M]^+^	336.2	292.2	50	70
Jatrorrhizine	[M]^+^	338.1	322.1	60	40
Magnoflorine	[M]^+^	342.2	297.07	60	26
Palmatine	[M]^+^	352.2	336.2	40	39
Tetrahydropalmatine (IS)	[M + H]^+^	356.26	192.11	115	35
6-Chloroflavone (IS)	[M + H]^+^	257.09	154.98	140	38

**Table 2:** Regression Equations, Linear Range, and LLOQs for 16 Analytes^a^

**Analytes**	**Regression**	**R** ^**2**^	**Range** **(ng/mL)**	**LLOQ** **(ng/mL)**
Hesperidin	y = 0.75531x + 0.00521	0.991697306	93.75–60000	10
Naringin	y = 0.01672x – 0.00115	0.992633616	7.81–5000	7.5
Ginsenoside Rb1	y = 0.01457x + 0.00007	0.991338836	3.13–2000	3
Mangiferin	y = 0.01786x + 0.00086	0.990502658	1.56–1000	1.5
Timosaponin A3	y = 0.03651x + 0.00156	0.993650112	0.08–50	0.05
Aloe-emodin	y = 0.02655x – 0.00243	0.990522563	0.31–200	0.3
Chrysophanol	y = 0.03906x – 0.00363	0.993849486	3.91–2500	2
Neomangiferin	y = 0.01551x + 0.00070	0.99022401	6.25–4000	3
6-Gingerol	y = 0.04990x – 0.00212	0.990880885	7.81–5000	2.5
8-Gingerol	y = 0.01027x + 0.00738	0.990900794	3.13–2000	1.5
Berberine	y = 0.05017x + 0.00491	0.99804096	3.13–2000	1.5
Coptisine	y = 0.09359x + 0.02065	0.994328066	0.16–100	0.15
Epiberberine	y = 0.10586x + 0.00074	0.996483098	1.56–1000	1.5
Jatrorrhizine	y = 0.72768x + 0.00729	0.992832888	1.56–1000	1.5
Magnoflorine	y = 1.84975x + 0.00584	0.996642822	6.25–4000	2
Palmatine	y = 0.63949x + 0.06197	0.99104016	1.56–1000	1.5

a x: peak area; y: concentration.

Matrix effects for all analytes ranged from 87.49% to 113.54%, and extraction recoveries ranged from 88.67% to 112.31%, with all RSD values below 15% (Table S14). These results indicate an acceptable method reliability under the current experimental conditions. The intraday and interday accuracies (SDs) ranged from 2.16% to 13.98% and 0.3% to 14.01%, respectively (Table S14). The interday precision (relative standard deviation) for the three spiked concentrations ranged from 3.88% to 9.98%, 3.58% to 9.88%, and 3.15% to 9.57%, respectively. The results of stability tests indicated that all analytes had high stability in mouse plasma after being stored under different conditions (Table S15).

### 3.5 Comparison of the Concentration–Time Curves of 16 Analytes in NCD- and HFD-Fed Mice after Administration of the KYJZ Formula

After method validation, the 16 target analytes, which comprised four flavonoids, two triterpenoids, two anthraquinones, six alkaloids, and two phenolic acids, were quantified in plasma samples after KYJZ administration by using the optimized UPLC-MS/MS method. The precise pharmacokinetic profiles of the 16 analytes are presented in Figure 5. The detailed pharmacokinetic parameters are calculated and summarized in Table S16, whereas a simplified table containing four key parameters (AUC_0‑t_, *C*_max_, *T*_1/2_, and *T*_max_) is shown in Table 3.^51^

**Figure 5:**
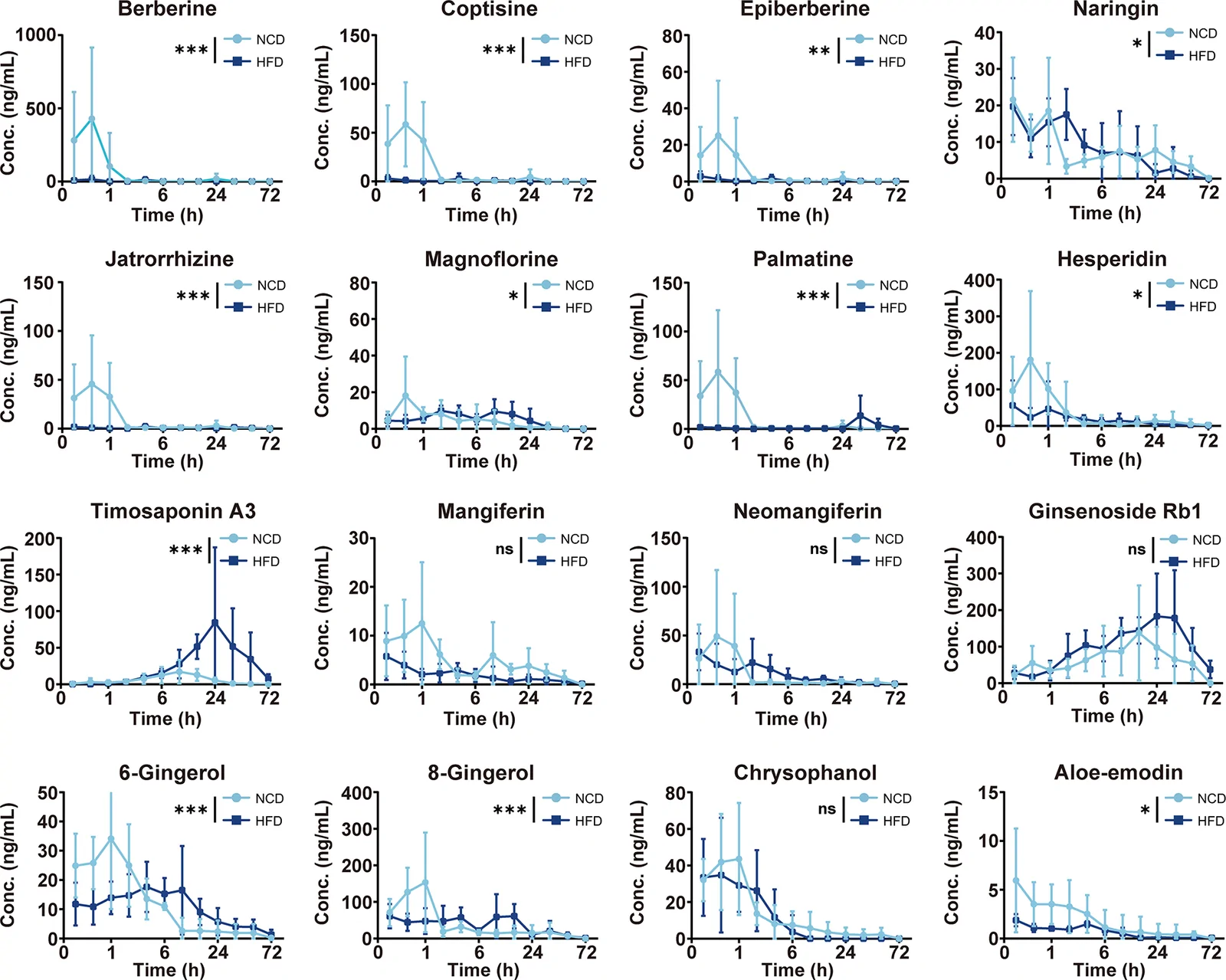
Mean plasma concentration–time profiles of 16 compounds in mice after oral administration of 2.1 g/kg KYJZ (mean ± SD, n = 6). Statistical significance relative to the NCD group is denoted by *P < 0.05, **P < 0.01, ***P < 0.001.

**Table 3:** Key Pharmacokinetic Parameters for 16 Analytes in NCD and HFD Mice after Oral Administration of KYJZ^a^

**Analytes**	**AUC**_**0–72h**_ **(h·ng/mL)**	**C**_**max**_ **(ng/mL)**	**T**1/2 (h)	**T**_**max**_ (h)
HFD-Hesperidin	439.24 ± 230.35	101.31 ± 69.53	16.35 ± 12.89	2.22 ± 2.95
NCD-Hesperidin	685.87 ± 1137.15	214.27 ± 177.6	17.56 ± 0	4.7 ± 9.48
HFD-Naringin	180.65 ± 151.8	24.29 ± 4.61	28.36 ± 28.47	2.75 ± 3.43
NCD-Naringin	288.07 ± 150.53	23.59 ± 10.9	557.18 ± 1297.96	0.5 ± 0.41
HFD-Ginsenoside Rb1	8508.16 ± 4312.43	235.29 ± 102.07	24.54 ± 7.54	19.67 ± 14.56
NCD-Ginsenoside Rb1	4064.23 ± 3040.81	160.23 ± 122.11	39.91 ± 29.84	8.42 ± 4.63
HFD-Mangiferin	63.06 ± 32.14**	7.23 ± 4.36	37.22 ± 62.46	1.59 ± 3.15
NCD-Mangiferin	162.29 ± 45.8	16.24 ± 10.13	39.12 ± 48.7	6.7 ± 9.56
HFD-Timosaponin A3	2900.5 ± 2515.27*	104.16 ± 90.01	15.32 ± 9.36	16 ± 6.2*
NCD-Timosaponin A3	292.89 ± 158.49	22.72 ±1 3.21	9.76 ± 5.76	8 ± 3.79
HFD-Aloe-emodin	12.69 ± 12.8	1.92 ± 0.58	35.84 ± 34.09	0.81 ± 1.56
NCD-Aloe-emodin	37.48 ± 48.24	6.34 ± 5.07	15.64 ± 1.73	0.42 ± 0.33
HFD-Chrysophanol	89.19 ± 55.63	42.99 ± 27.53	49.57 ± 66.72	0.86 ± 0.64
NCD-Chrysophanol	215.07 ± 217.9	54.44 ± 32.79	23.29 ± 18.28	0.7 ± 0.36
HFD-Neomangiferin	227.23 ± 108.3*	36.38 ± 22.74	19.98 ± 14.44	1.11 ± 1.59
NCD-Neomangiferin	161.16 ± 132.65	59.34 ± 74.65	20.07 ± 14.55	1.17 ± 1.4
HFD-6-Gingerol	427.02 ± 112.44*	26.74 ± 11.71	25.48 ± 22.92	2.7 ± 2.91
NCD-6-Gingerol	226.02 ± 164.53	41.17 ± 20.48	21.16 ± 28.31	0.95 ± 0.62
HFD-8-Gingerol	1327.35 ± 392.97	107.59 ± 41.49	14.63 ± 7.84	4.36 ± 3.16*
NCD-8-Gingerol	914.61 ± 440.09	199.08 ± 106.99	68.65 ± 79.85	0.7 ± 0.36
HFD-Berberine	29.09 ± 33.28**	17.91 ± 21.99*	-	0.28 ± 0.17
NCD-Berberine	404.87 ± 176.89	567.4 ± 424.94	-	8.36 ± 12.12
HFD-Coptisine	12.85 ± 14.23**	5.22 ± 4.65**	4.63 ± 1.86	4.06 ± 3.5
NCD-Coptisine	113.91 ± 64.6	66.95 ± 44.2	4.13 ± 6.22	4.45 ± 9.58
HFD-Epiberberine	10.87 ± 7.65**	3.52 ± 2.49*	30.04 ± 20.01	7.5 ± 14.3
NCD-Epiberberine	54.6 ± 29.16	30.41 ± 28.35	10.01 ± 6.36	4.83 ± 9.41
HFD-Jatrorrhizine	38.04 ± 22.44*	3.34 ± 1.93*	23.45 ± 1.14	4.06 ± 3.5
NCD-Jatrorrhizine	94.39 ± 43.67	50.03 ± 48.78	4.77 ± 3.31	8.5 ± 12.01
HFD-Magnoflorine	87.82 ± 69.63	6.59 ± 2.25	5.22 ± 4.44	7.33 ± 4.5**
NCD-Magnoflorine	36.84 ± 35.46	11.87 ± 8.72	5.25 ± 5.11	0.7 ± 0.65
HFD-Palmatine	765.27 ± 522.12	66.49 ± 77.29	10.51 ± 0	32.08 ± 16.2**
NCD-Palmatine	454.68 ± 177.4	248.33 ± 248.72	31.28 ± 54.55	4.58 ± 9.52

a NCD: normal chow diet; HFD: high-fat diet; significance relative to the NCD group is indicated as follows: *P < 0.05, **P < 0.01, ***P < 0.001.

The pharmacokinetic study revealed distinct differences in drug exposure, absorption, and elimination between the HFD and NCD groups across the multiple compounds. In the NCD group, alkaloids (coptisine, epiberberine, jatrorrhizine, magnoflorine, palmatine), flavonoids (naringin, hesperidin), and anthraquinones (chrysophanol, aloe-emodin) exhibited rapid absorption, all achieving *C*_max_ at an earlier *T*_max_, indicating swift absorption in the gastrointestinal tract following oral KYJZ administration.^52–54^ Conversely, ginsenoside Rb1, a triterpenoid, exhibited slower absorption, consistent with previous findings on its pharmacokinetic profile.^55^ The *T*_max_ values of timosaponin A3, 6-gingerol, and 8-gingerol were delayed compared with those reported in previous studies, potentially influenced by different herb combinations.^25,56,57^ *t*_1/2_ values varied substantially. For instance, *t*_1/2_ was prolonged for flavonoids but shortened for triterpenoids in the NCD group. Pharmacokinetic profiles differed significantly between the HFD and the NCD groups. Flavonoids (naringin, hesperidin), anthraquinones (chrysophanol, aloe-emodin), and particularly protoberberine-type alkaloids (berberine, coptisine, epiberberine, jatrorrhizine) exhibited lower AUC_0‑t_ in the HFD group, whereas triterpenoids (ginsenoside Rb1, timosaponin A3) and phenolic acids (6-gingerol, 8-gingerol) displayed higher AUC_0‑t_ in the HFD group. Interestingly, 6-gingerol and 8-gingerol displayed a lower *C*_max_ in the HFD group, suggesting complex absorption dynamics. These findings indicate that obesity could increase the bioavailability of hydrophobic compounds, such as triterpenoids and phenolic acids, specifically because of the higher fat content in the HFD group. These variations might affect the absorption and tissue distribution of the circulating components. Additionally, magnoflorine and 8-gingerol displayed biphasic peaks, potentially indicating drug redistribution between different tissues.^53^ Notably, the plasma concentration of timosaponin A3 was significantly increased, indicating potential tissue accumulation and significance as a pharmacodynamically active ingredient.

However, pharmacokinetic parameters such as *t*_1/2_ of berberine could not be determined because its plasma concentrations were below the LLOQ in the majority of samples. This finding is consistent with its extremely low oral bioavailability in rats, which is largely attributable to extensive intestinal first-pass elimination.^58^

### 3.6 Tissue Distribution of 16 Analytes in the KYJZ Formula in HFD-Fed Mice

The distribution and content of the target compounds in mouse tissues (heart, liver, spleen, lungs, and kidneys) were investigated after oral KYJZ administration (2.1 g/kg), and the results are presented in Figure 6. At 0.5 h after oral administration, the target compounds were detected in all tissues of mice, indicating that these compounds were rapidly absorbed and distributed. The concentrations of flavonoids, phenolic acids, and alkaloids decreased significantly from 2 to 6 h, suggesting no significant accumulation in the tissues. Triterpenoids, although also rapidly distributed to the tissues, exhibited higher concentrations at 6–12 h, indicating slow absorption and possible accumulation in the tissues. The levels of 14 of the 16 target analytes (berberine, coptisine, epiberberine, jatrorrhizine, magnoflorine, palmatine, naringin, hesperidin, timosaponin A3, neomangiferin, 6-gingerol, 8-gingerol, chrysophanol, and aloe-emodin) were greater in the liver than in the other four tissues, emphasizing the importance of the liver in absorbing and metabolizing drugs. In particular, protoberberine-type alkaloids, including berberine, coptisine, epiberberine, jatrorrhizine, and palmatine, showed high abundance in the liver but low AUC_0‑t_ in plasma, suggesting preferential hepatic distribution of these compounds. This pattern is consistent with the well-characterized role of OCT1 and hepatic uptake transporters in concentrating quaternary alkaloids within hepatocytes,^59^ and with the established hepatic first-pass extraction of protoberberine alkaloids observed in multiple species.^60^ Given that KYJZ is clinically indicated for metabolic disorders involving hepatic pathology, the liver may represent a relevant target organ for its bioactive constituents. This notion is further supported by a study on the *Curcuma zedoaria*, *Atractylodes macrocephala* herb pair, which demonstrated that hepatic accumulation of active components was associated with enhanced antichronic liver injury efficacy.^61^ Taken together, our findings suggest that tissue-specific accumulation of active compounds may be associated with their pharmacological effects.

**Figure 6:**
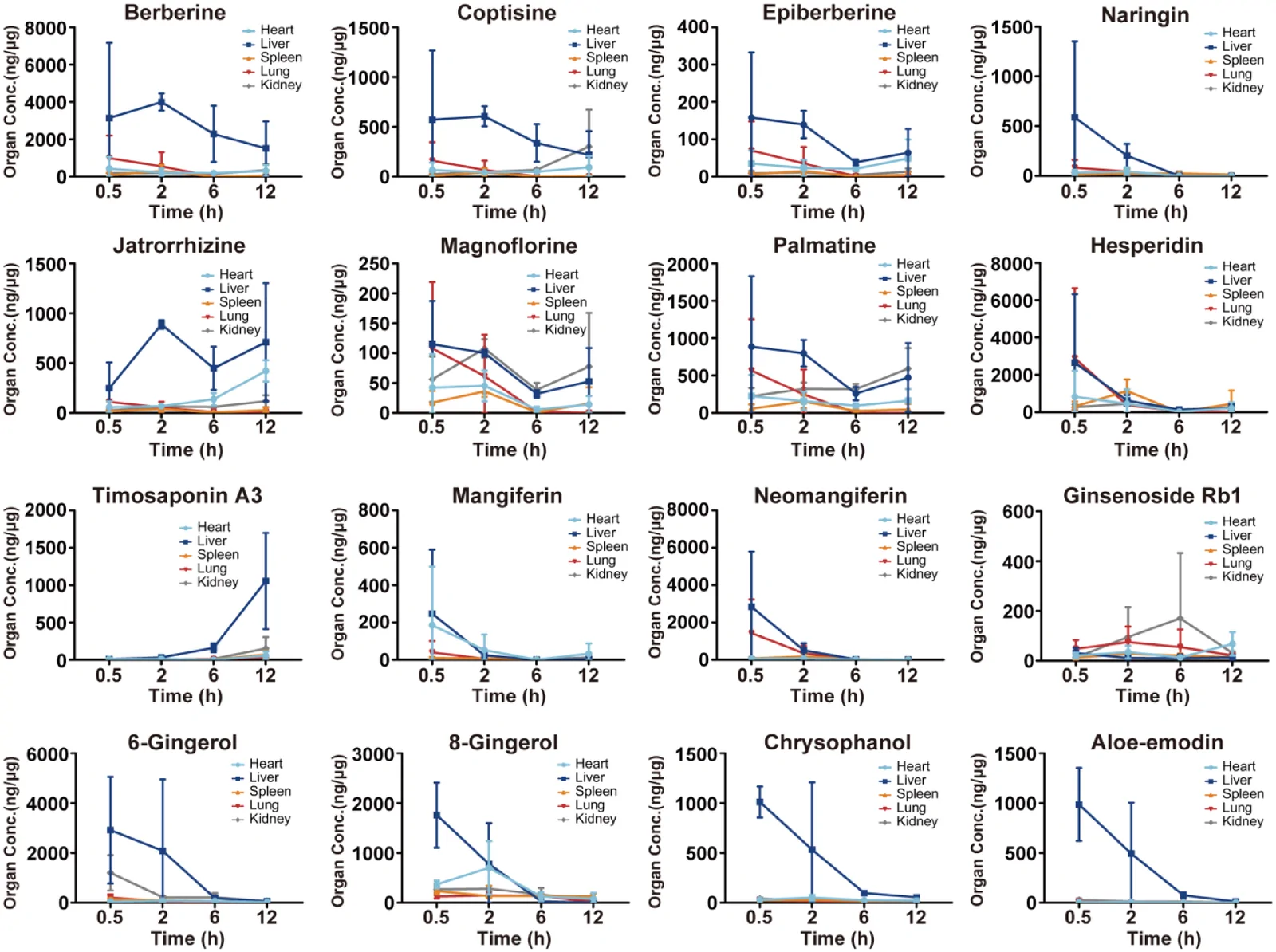
Mean organ (heart, liver, spleen, lung, kidney) concentration–time profiles of 16 compounds in mice after oral administration of 2.1 g/kg KYJZ (mean ± SD, n = 3).

However, we did not perform direct pharmacodynamic assays to confirm their biological activity in the liver, such as hepatic lipid profiling, target protein modulation, or liver-specific functional readouts. In addition, the tissue distribution study was limited by a small sample size (3 per time point) and the absence of an NCD control group. Consequently, small differences among tissues should not be overinterpreted, and the extent to which the observed hepatic accumulation is specific to the obese state remains to be determined. Thus, our conclusion regarding the hepatic relevance of these compounds is based on pharmacokinetic tissue distribution data and awaits validation in future pharmacological and tissue distribution studies with larger cohorts and NCD control groups.

## 4 Conclusions

The “chemical profile–circulating components–*in silico* screening–pharmacokinetic behavior–tissue distribution” pipeline offers a strategy for mining the potential bioactive constituents from TCM. We demonstrated the power of 2D-LC–MS for comprehensive chemical profiling of KYJZ and further identified its circulating components. Using *in silico* screening, 16 potential bioactive components were identified. Our findings revealed that the pharmacokinetics of these components are fundamentally altered in a diet-induced obese state. However, most of these components, including protoberberine-type alkaloids, are poorly absorbed into the bloodstream but highly distributed in the liver, indicating preferential hepatic accumulation of these compounds. Whether this tissue-level distribution translates into hepatic pharmacodynamic activity relevant to the antiobesity effects of KYJZ requires direct functional validation in future studies. This work provides both a strong scientific rationale for applying multidimensional chromatography to characterize the chemical basis of KYJZ and establishes a novel pipeline for investigating TCMs.

## Supplementary Material





## Data Availability

All relevant data are within the manuscript and its additional files.

## Notes

Detailed information for tissue sample preparation and method validation; optimization of two-dimensional chromatography; evaluation of orthogonality; identification and classification of the circulating components; supporting information for pharmacokinetic method validation (DOCX)
The detailed information on KYJZ’s compositions; standard compounds and columns; details of the 469 MRM^HR^ transitions, retention times, and normalized retention times for all columns; orthogonal coefficients of different column pairs; chemical profiling of the Kaiyu Jiangzhuo formula; *in silico* docking and ranking of circulating components against antiobesity targets; accuracy, precision, recovery, matrix effect, and stability for the determination of 16 analytes in mouse plasma samples (XLSX)

## Abbreviations

KYJZ: Kaiyu Jiangzhuo
TCM: Traditional Chinese medicine
2D-LC–MS: Two-dimensional liquid chromatography–mass spectrometry
^1^D: First-dimensional
^2^D: Second-dimensional
UPLC: Ultraperformance liquid chromatography
DDA: Data dependent acquisition
MRM^HR^: High-resolution multiple-reaction monitoring
NCD: Normal chow diet
HFD: High fat diet
IEC: Ion exchange chromatography
SCX: Strong cation exchange
SAX: Strong anion exchange
Q-TOF: Quadrupole time-of-flight
LLOQ: Lower limit of quantification
