# Supplementary material for: Deciphering the Material Basis of an Herbal Formula via Integrated Two-dimensional Chromatography, In Silico Screening, and Comparative Pharmacokinetics: A Case Study of Kaiyu Jiangzhuo Formula
Source: ACS Omega. 2026 Oct 29;11(44):69287–301. doi: 10.1021/acsomega.6c09515 (PMC13756683; doi:10.1021/acsomega.6c09515)
Supplement: Supplementary file 1 [file ao6c09515_si_001.docx]

**Deciphering the Material Basis of an Herbal Formula via Integrated Two-dimensional Chromatography, In Silico Screening, and Comparative Pharmacokinetics: A Case Study of Kaiyu Jiangzhuo Formula**

Nanqi Hou^a,b^, Qinjing Xia^b^, Zuoyang Li^b^, Xinyue Cao^b^, Zichen Tian^a^, Yuying Zhou^a^, Zepeng Zhang^c^, Kai Yin^a^, Shilei Wang^b^, Dandan Si^d^, Zhuo Man^d^, Xiaolin Tong^b,e*^, Hang Su^a,b*^, Xiangyan Li^a,b*^

^a^Department of Endocrinology, The First Affiliated Hospital of Changchun University of Chinese Medicine, Changchun, 130021, China.

^b^Northeast Asia Research Institute of Traditional Chinese Medicine, Key Laboratory of Active Substances and Biological Mechanisms of Ginseng Efficacy, Ministry of Education, Jilin Provincial Key Laboratory of Bio-Macromolecules of Chinese Medicine, Changchun University of Chinese Medicine, Changchun, 130117, China.

^c^Research Center of Traditional Chinese Medicine, The First Affiliated Hospital of Changchun University of Chinese Medicine, Changchun, Jilin, 130021, China.

^d^Sciex China, Beijing, 100015, China.

^e^Institute of Metabolic Diseases, Guang'an men Hospital, China Academy of Chinese Medical Sciences, Beijing, 100053, China.

*Corresponding authors. E-mail address: Xiangyan Li, [xiangyan_li1981@163.com;](mailto:xiangyan_li1981@163.com;) Hang Su, suhang0720[@live.cn](mailto:suhang0720@live.cn); Xiaolin Tong, [tongxiaolin@vip.163.com](mailto:tongxiaolin@vip.163.com).

**Methods**

1. Supporting information for tissue sample preparation

A 20 mg of tissue samples were accurately weighed and five times the weight of normal saline was added, followed by three times of homogenization at 5,000 rpm for 15 sec. Subsequently, four times the volume of methanol: acetonitrile (v/v=1:1, Tetrahydropalmatine, 50 ng/mL and 6-chloroflavone, 5 ng/mL ) was added and the mixture was vortexed for 1 min. The following operations were the same as section 2.4.

2. Supporting information for method validation

Selectivity was assessed by comparing the chromatograms of six blank plasma with those of six blank plasma spiked with the target analytes. Calibration curves were made by graphing a linear regression equation with 1/x^2^ weighting, which versus the peak area ratio of the analyte and internal standard with the concentration of the standard, and the correlation coefficient for each calibration curve should be higher than 0.99. The lower limit of quantification (LLOQ) for analytes were determined by the lowest concentration which had a signal-to-noise ratio greater than 10. Accuracy and precision were assessed by analyzing the QC of low, medium, and high concentrations (n=6) in one day (intra-day) and over three consecutive days (inter-day), and the relative standard deviation and relative standard deviation should be no more than 15%, which is acceptable for the accuracy and precision of intra-day and inter-day. The stability of target analytes in mice plasma was assessed by analyzing QC at low, medium, and high concentrations in different storage environments: room temperature for 24h, 4℃ for 24h, and 80℃ for 14 days. Matrix effects were investigated by comparing the peak area ratios of each analyte versus the internal standard which were extracted from QC samples and extracted neat solution spiked with standards at the same concentration. Recoveries were obtained by comparing the peak area ratios of each analyte versus the internal standard which were extracted from QC samples and post-extracted blank mice plasma spiked with standards at the same concentration.

**Figures**

**Figure S1:** Supplementary material for optimization of two-dimensional chromatography. Optimization of first dimensional chromatography (A-D) and comparison of second dimensional chromatographic columns (E-J).

**Figure S2:** The scatter plots for 26 components determined on 8 pairs of candidate column combinations displaying their separation difference.

**Figure S3:** Comparison of the number of circulating components identified by two mass spectrometry methods (A). Classification of the identified circulating components (B).

**Figure S4:** Heavy-atom RMSD between redocked and crystallographic poses for the ten co-crystallized ligands (dashed line: 2.0 Å success criterion).

**Figure S5:** Threshold sensitivity analysis. Bars: number of compounds selected at each Composite Z-score threshold; lines: retention of the original 16 candidates and of nine independently reported active constituents.

**Figure S6:** Correlations among docking metrics and robustness of compound rankings under alternative weighting schemes. Pairwise Spearman correlations among the three docking metrics (n = 990 ligand–target pairs) (A). Spearman correlation of compound rankings under alternative weighting schemes versus the original equal-weight ranking (labels: ρ and top-16 overlap) (B).

**Figure S7:** Supplementary materials for pharmacokinetic method validation.
